# Relative Dose Intensity and Pathologic Response Rates in Patients With Breast Cancer and With and Without HIV Who Received Neoadjuvant Chemotherapy

**DOI:** 10.1200/GO.22.00016

**Published:** 2022-05-18

**Authors:** Yehoda M. Martei, Mohan Narasimhamurthy, Dipho I. Setlhako, Gezahen Ayane, Tlotlo Ralefala, Sebathu Chiyapo, Robert Gross, Lawrence N. Shulman, Surbhi Grover, Angela DeMichele

**Affiliations:** ^1^ Department of Medicine (Hematology-Oncology), University of Pennsylvania, Philadelphia, PA; ^2^ Botswana University of Pennsylvania Partnership, Gaborone, Botswana; ^3^ Department of Pathology, University of Pennsylvania, Philadelphia, PA; ^4^ Princess Marina Hospital, Gaborone, Botswana; ^5^ Gaborone Private Hospital, Gaborone, Botswana; ^6^ University of Pennsylvania, Department of Medicine (Infectious Diseases), Philadelphia, PA; ^7^ Department of Radiation Oncology, University of Pennsylvania, Philadelphia, PA

## Abstract

Patients who are HIV-positive and have breast cancer have worse overall survival (OS) compared with patients who are HIV-negative. Pathologic complete response (pCR) and relative dose intensity (RDI) of chemotherapy are associated with survival. We assessed whether pCR and RDI rates were lower for patients who are HIV-positive and received neoadjuvant chemotherapy (NACT).

**METHODS:**

This was a prospective cohort analysis of patients initiating NACT in Botswana (February 2017 to September 2019). Primary outcomes were pCR and RDI; secondary outcomes were OS and toxicity. HIV status and zidovudine (ZDV) treatment were stratification factors. Multivariable analysis was used to control for confounding.

**RESULTS:**

In total, 26 of 110 enrolled individuals were HIV-positive. In univariable analysis, HIV-positive (odds ratio [OR] = 0.2; *P* = .048) and RDI < 0.85 (OR = 0.30; *P* = .025) were associated with pCR. In multivariable analysis, the magnitude of association decreased for HIV-positive (OR = 0.28; *P* = .11), but RDI < 0.85 remained independently associated with pCR (OR = 0.32; *P* = .035). Patients who are HIV-positive had significantly lower mean RDI, and those on ZDV had significantly lower RDI. Ninety-one (83%) were stage III with 2-year OS significantly worse for patients who are HIV-positive (58% *v* 74%). Hazard ratio for all-cause mortality was 2.68 (95% CI, 1.17 to 6.13; *P* = .028) in patients who are HIV-positive compared with patients who are HIV-negative. Toxicity rates were similar despite patients who are HIV-positive receiving significantly lower dose intensity chemotherapy.

**CONCLUSION:**

Patients who are HIV-positive and have breast cancer in Botswana have lower pCR rates and also receive lower dose intensity therapy, which may contribute to worse OS. Patients who are HIV-positive on ZDV-containing regimens received even lower dose intensity of NACT. Administering optimal dose intensity in patients who are HIV-positive remains a challenge, and targeted interventions that address modifiable risk factors are needed to improve therapy delivery and outcomes.

## INTRODUCTION

Antiretroviral therapy (ART) has led a drop in overall incidence of AIDS-defining cancers and an upsurge in age-related non–AIDS-defining cancers, as the HIV population ages.^1,2^ Women living with HIV (WLWH) are not at increased risk of breast cancer compared with women who are HIV-negative^2^; however, they have been observed to have higher mortality in numerous settings.^3–5^ A large retrospective analysis of US-based cancer cohorts and a meta-analysis which included 14 studies from sub-Saharan Africa (SSA) have shown an increased risk of all-cause and breast cancer–specific mortality in patients who are HIV-positive compared with patients who are HIV-negative.^3,4^ The underlying mechanisms for the observed survival disparities arepoorly understood^6^ because previous large retrospective analysis has been limited by lack of detailed treatment delivery and toxicity data. There are also limited data on association of treatment toxicity and adequacy of cancer therapy delivery with HIV viremia or ART regimen. Botswana is a middle-income country in SSA with the fourth highest prevalence of HIV in the world.^7^ Breast cancer represents 18% of all cancers diagnosed and 12.5% of cancer-associated deaths with an age-standardized mortality rate of 7.0 per 100,000.^8^ Additionally, 25%-30% of patients with breast cancer are WLWH.^9^ Recent data from the region suggest worse survival in WLWH with breast cancer compared with patients who are HIV-negative,^5^ which represents a huge public health burden.

**CONTEXT**

**Key Objective**
To evaluate survival outcomes in patients living with HIV and patients who are HIV-negative and have breast cancer in Botswana and furthermore to assess whether patients who are HIV-positive receive lower relative dose intensity (RDI) of neoadjuvant chemotherapy and have lower pathologic complete response rates.
**Knowledge Generated**
Patients who are HIV-positive received significantly lower RDI of chemotherapy, with lowest RDI in patients on zidovudine-based antiretroviral regimens. Furthermore, patients who are HIV-positive had lower pathologic complete response rates. These observed differences in our prospective cohort may partly account for survival disparities between patients who are HIV-positive and HIV-negative and who are diagnosed with breast cancer.
**Relevance**
Administering optimal dose intensity chemotherapy in patients with HIV and who have breast cancer remains a challenge and adversely affects survival. Care integration with HIV specialists and pharmacists should be considered to address modifiable risk factors.

Response to neoadjuvant chemotherapy (NACT), as measured by the pathologic complete response (pCR), is a strong predictor of event-free survival (EFS) within specific molecular subtypes.^10–12^ Relative dose intensity (RDI) is a composite measure that includes actual cumulative dose of chemotherapy received and duration of treatment received, expressed as a proportion of the intended standard dose and duration of therapy. RDI < 0.85 has been associated with worse survival in patients with nonmetastatic breast cancer.^13–15^ We hypothesized that patients who are HIV-positive in Botswana might receive lower RDI that may have an adverse impact on pCR rates and subsequent survival. Therefore, our aim was to assess whether pCR rates were lower for patients who are HIV-positive and received NACT compared with patients who are HIV-negative and whether lower chemotherapy RDI was different between WLWH and patients who are HIV-negative diagnosed with breast cancer. We also evaluated overall survival (OS) and toxicity in patients who were HIV-positive versus HIV-negative receiving NACT as secondary outcomes.

## METHODS

### Study Design and Participants

This was a prospective cohort study of patients who are HIV-positive or HIV-negative with newly diagnosed breast cancer, age ≥ 18 years, and with stage I-III disease who initiated NACT at Princess Marina Hospital, the largest cancer care referral center in Botswana. Patients who were pregnant, had previously undergone excisional biopsy, received prior chemotherapy, or unable to consent were excluded. Informed consent was obtained before enrollment. This study was approved by the Institutional Review Boards at the University of Pennsylvania, the Botswana Human Research Development Committee, Ministry of Health, and Princess Marina Hospital.

### Treatment

Guidelines for breast cancer NACT treatment in Botswana is aligned with the National Comprehensive Cancer Network harmonized guidelines for enhanced resource countries,^16^ which consists of doxorubicin 60 mg/m^2^ and cyclophosphamide 600 mg/m^2^ once a day, every 21 days for four cycles, followed by paclitaxel 175 mg/m^2^ once a day, every 21 days for four cycles (doxorubicin plus cyclophosphamide, followed by paclitaxel [AC-T]); plus trastuzumab 6 mg/kg once a day every 21 days, with a loading dose of 8 mg/kg for patients with human epidermal growth factor receptor 2 (HER2)—positive tumors (AC-TH). Primary prophylaxis with granulocyte colony-stimulating factor is not administered in patients receiving AC-T. If operable, patients are recommended to undergo modified radical mastectomy (approximately 4-6 weeks after the last cycle of chemotherapy) with pathology evaluation of response of surgical specimen. Breast conservation surgery is rarely offered because of lack of surgical expertise and image-guided resources. All pathology assessments were performed using standardized criteria at the Botswana National Health Laboratory. Patients with hormone receptor-positive disease and HER2-positive disease were prescribed endocrine therapy and trastuzumab, respectively.

### Data Collection and Assessment

Baseline patient and clinical variables collected included demographics, cancer stage, molecular subtype, HIV status, and CD4 count at the initiation of breast cancer therapy and ART for patients who are HIV-positive. Chemotherapy dosing and date, laboratory data, and toxicity were assessed at each cycle visit. Toxicity, including laboratory assessment of complete blood count and creatinine and total bilirubin level, and febrile neutropenia, peripheral neuropathy, and pain were assessed using common toxicity criteria 4.03, the most recent version at the start of the study.^17^ Toxicities were assessed at each chemotherapy cycle visit, at their surgery appointment, and every three-months follow-up conducted after treatment via phone or at clinic visit to assess vital status until March 2, 2021.

### Primary Outcome and Exposure

Our primary outcomes were RDI and pCR. Since RDI < 0.85 for adjuvant chemotherapy regimens has been associated with inferior disease-free survival and OS in patients with breast cancer,^14^ we used this as a cutoff for suboptimal therapy receipt. We also assessed RDI as a continuous variable and compared means of the RDI stratified by HIV status and treatment. pCR was defined as no residual invasive cancer in the breast and lymph nodes, with noninvasive residual disease in breast allowed,^12^ and was analyzed overall for the group and by receptor status—classified as triple-negative, HER2-positive, or hormone receptor-positive/HER2-negative. Secondary outcomes were toxicity and OS by HIV status. OS was defined as time from initiation of treatment until death from any cause.

### Statistical Analysis

Chi-squared (χ^2^) or Fisher's exact tests (when expected cell size was < 5) were used to compare proportions of categorical patient baseline, tumor, and treatment characteristics in patients who are HIV-positive and HIV-negative. Student's *t*-tests were used to compare means of normally distributed continuous variables, and Wilcoxon rank-sum tests were used to compare medians of continuous variables with nonparametric distributions. The OS analyses were performed using the Kaplan‐Meier estimator and limited to stage III patients who were the majority of patients, given the wide variation in breast cancer survival by stage.^18^ Comparisons of OS between patients who were HIV-positive and HIV-negative were analyzed using log‐rank tests and crude hazard ratios (HRs) and corresponding 95% CIs, computed using Cox proportional hazards regression. Within the population who are HIV-positive, two further subcohorts were created on the basis of whether the ART regimen was zidovudine (ZDV)-containing versus non–ZDV-containing, since ZDV is known to cause myelosuppression.^19^ RDI analyses were repeated by using the ZDV regimen. Grade ≥ 3 toxicity rates were compared by HIV status and ZDV regimen. Multivariable linear and logistic regressions were used to control for potential confounding variables for RDI and pCR, respectively. Covariates significantly associated with exposure and outcome with *P* < .2 in the univariable or bivariable analyses were included in the multivariable model. For pCR analysis, hormone receptor status was forced in the model given the known association with pCR in the literature.^10^ Analyses were carried out in STATA version 16 (StataCorp, College Station, TX). *P* values for pCR analyses were a priori one-sided given the aim to test only if rates were lower for HIV-positive than HIV-negative; all other *P* values were two-sided.

## RESULTS

**FIG 1:**
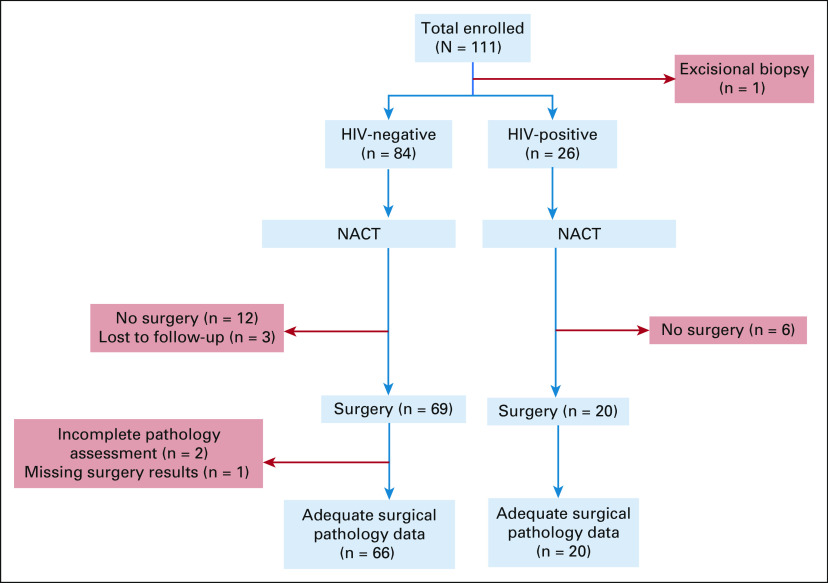
STROBE flow diagram of the prospective cohort study of patients who are HIV-positive and HIV-negative and have breast cancer who received NACT at Princess Marina Hospital. NACT, neoadjuvant chemotherapy.

One hundred eleven eligible patients with breast cancer were prospectively enrolled from February 2017 to September 2019. One patient was excluded after enrollment because she had an excisional biopsy, resulting in 110 patients, including 26 (24%) with HIV in this analysis (Fig 1). Patients who are HIV-positive were significantly younger at diagnosis, less likely to be overweight or obese, and more likely to have hormone receptor-positive disease (Table 1). All but one patient who is HIV-positive was receiving ART (Table 1). Nine patients who are HIV-positive (35%) were on ZDV-containing treatment regimens (Table 1).

**TABLE 1:**
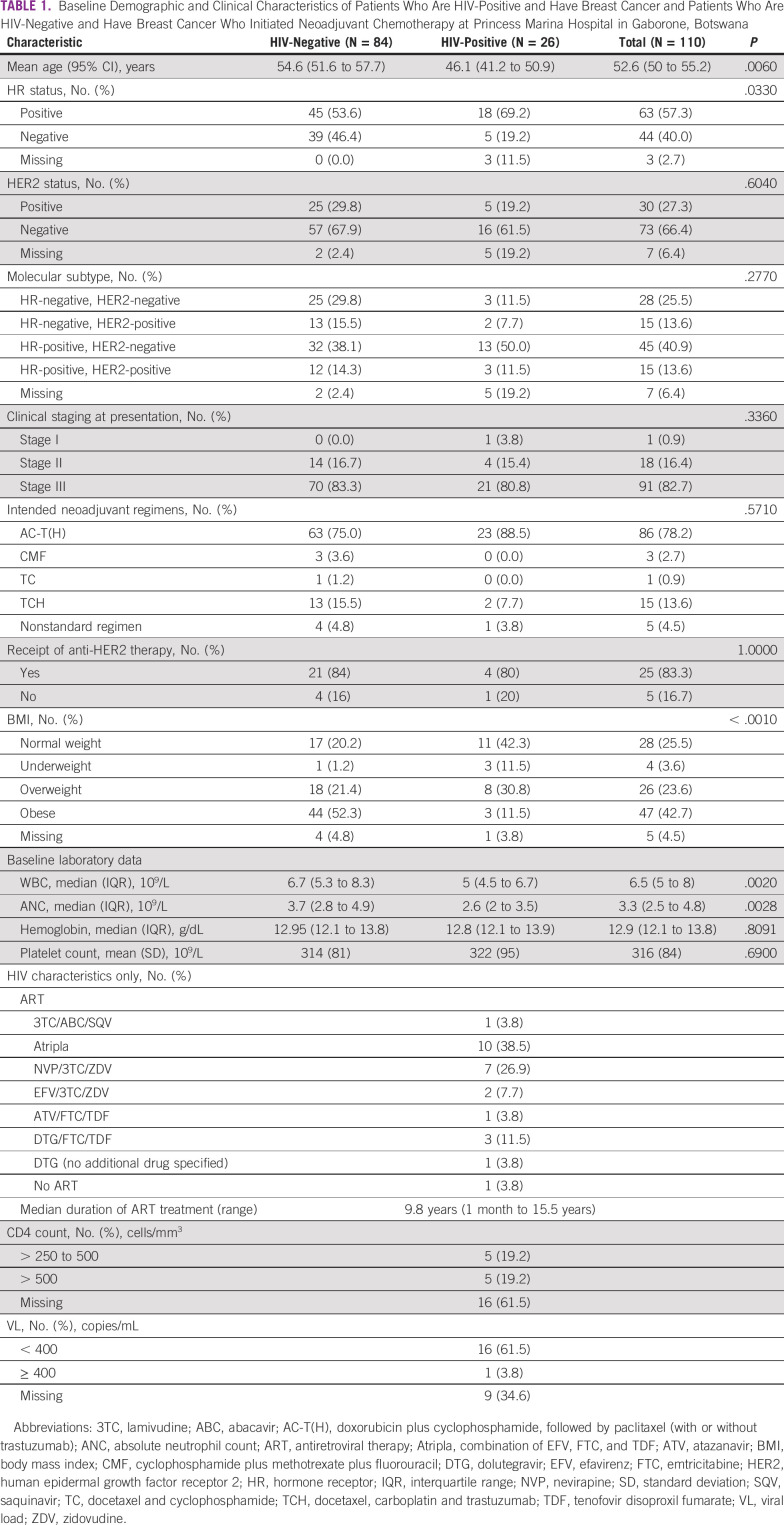
Baseline Demographic and Clinical Characteristics of Patients Who Are HIV-Positive and Have Breast Cancer and Patients Who Are HIV-Negative and Have Breast Cancer Who Initiated Neoadjuvant Chemotherapy at Princess Marina Hospital in Gaborone, Botswana

### Pathologic Complete Response

Of the patients who underwent surgery and had adequate surgical pathology data (n = 86), the pCR rate was significantly lower among patients who are HIV-positive compared with patients who are HIV-negative (5% [1/20] *v* 21% [14/52]; odds ratio [OR] = 0.20; one-sided *P* = .048). As expected, pCR rates by receptor status were highest overall in HER2-positive disease, followed by triple-negative disease and hormone receptor-positive/HER2-negative disease (Table 2). In the univariable analysis, RDI < 0.85 was associated with pCR (OR = 0.3; one-sided *P* = .03), but age at diagnosis, hormone receptor status, and BMI were not associated with pCR with *P* < .2 threshold. In the multivariable analysis, including RDI and hormone receptor status forced into the model, HIV-positive (OR = 0.28; one-sided *P* = 0.11) was not significantly associated with pCR, but RDI < 0.85 (OR = 0.32; one-sided *P* = 0.035) was significantly associated with pCR. In the adjusted model, hormone receptor‐positive status was associated with a lower likelihood of pCR, although the association was not significant in our cohort (OR=0.79; one-sided *P* = 0.31).

**TABLE 2:**
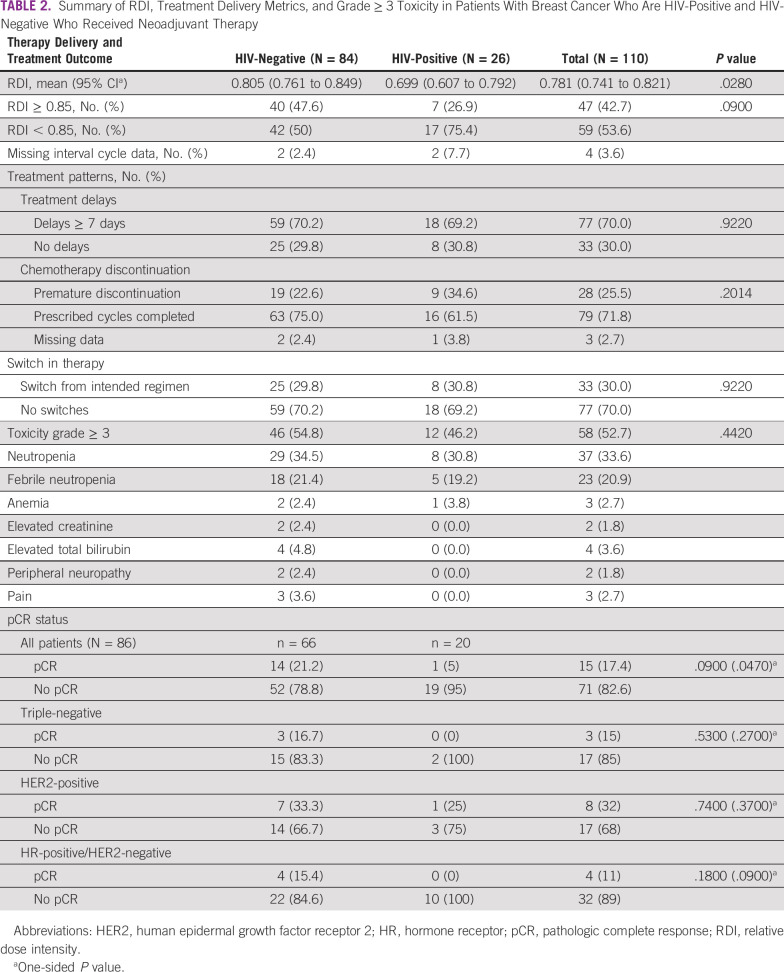
Summary of RDI, Treatment Delivery Metrics, and Grade ≥ 3 Toxicity in Patients With Breast Cancer Who Are HIV-Positive and HIV-Negative Who Received Neoadjuvant Therapy

### Treatment Adequacy

Patients who are HIV-positive received a significantly lower mean RDI of NACT compared with patients who are HIV-negative (0.70 [95% CI, 0.61 to 0.792] *v* 0.805 [95% CI, 0.761 to 0.849], *P* = .028; Table 2). Approximately half of patients who are HIV-negative (40 of 84) received neoadjuvant treatment with RDI ≥ 0.85 compared with less than one-third (7 of 26) in the HIV-positive group (Table 2). Among patients who are HIV-positive, those taking ZDV-containing regimens received a lower mean RDI of 0.58 (95% CI, 0.38 to 0.77) compared with RDI = 0.77 (95% CI, 0.68 to 0.86) in patients taking non–ZDV-containing regimen (*P* = .0325). Additionally, 78% (7 of 9) of patients on ZDV-containing regimen had RDI < 0.85 compared with 67% (10 of 15) of patients on non–ZDV-containing regimen (*P* = .669).

In the simple linear regression model, HIV status (β = –0.11, *P* = .028) significantly predicted RDI. Being underweight was associated with RDI (β = –0.14, *P* = .193) and was included in the multiple linear regression model. In the adjusted model, HIV remained significantly associated with RDI (β = –0.12, *P* = .015).

### Treatment Patterns and Toxicity

Despite lower RDI in patients who are HIV-positive, rates of grade ≥ 3 toxicity were similar in both groups, most commonly because of neutropenia or febrile neutropenia. The summary of other grade ≥ 3 toxicity assessed by Common Terminology Criteria for Adverse Events criteria is provided in Table 2. We found a higher proportion of early discontinuation of systemic chemotherapy in the HIV-positive group compared with the HIV-negative group (35% [9/26] *v* 23% [19/84], *P* = .20); however, this difference was not statistically significant, and overall, treatment discontinuation was high in both cohorts (Table 2). A detailed summary of chemotherapy administration data showed that the proportion of cycles with dose reductions in the first cycle was 14% (12 of 84) for the HIV-negative group compared with 24% (6 of 26) for the HIV-positive group (*P* = .359): 25% (4 of 16) for the non-ZDV group and 22% (2 of 9) for the ZDV group (*P* = 1.00). The proportion of patients with any dose reduction during treatment was 21% (18 of 84) for the HIV-negative group, compared with 32% (8 of 26) for the HIV-positive group (*P* = .184): 31% (5 of 16) for the non-ZDV group and 33% (3 of 9) for the ZDV group (*P* = .626). The mean duration of a cycle for patients who are HIV-negative was 27 days (95% CI, 26 to 29; median 25 days) and it was 31 days for those who are HIV-positive (95% CI, 27 to 35; median 28 days; *P* = .087): 28 days for the non-ZDV group (95% CI, 24 to 32; median 27) and 35 days for the ZDV group (95% CI, 25 to 45; median 31 days; *P* = .091).

### Survival Outcomes

**FIG 2:**
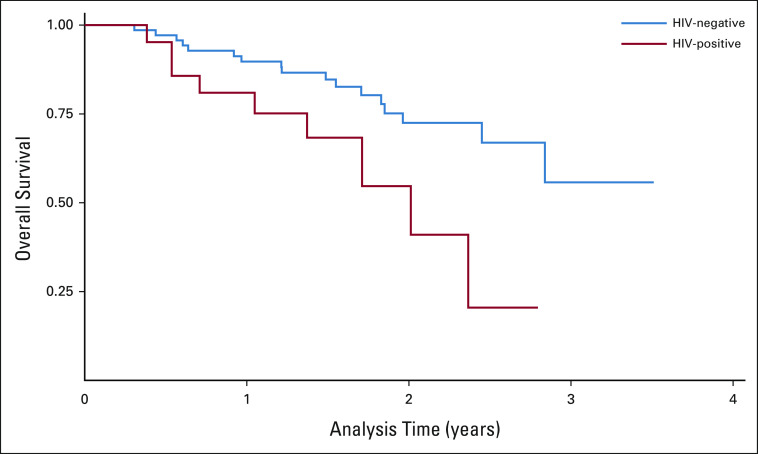
Kaplan-Meier overall survival curves for patients who are HIV-positive and HIV-negative and have breast cancer. Survival curves showing cumulative survival differences between patients who are HIV-positive and HIV-negative with stage III breast cancer.

After a median follow-up of approximately 20 months, the estimated 2-year OS for stage III patients was significantly lower in patients who are HIV-positive and have breast cancer compared with patients who are HIV-negative and have breast cancer (58% *v* 74%, respectively; Fig 2), with an unadjusted HR for death from any cause of 2.7 (95% CI, 1.2 to 6.1; *P* = .028) for HIV-positive compared with HIV-negative. A similar trend of worse OS in the patient group who are HIV-positive and have breast cancer was observed for the combined cohort and subset of patients with hormone receptor-positive/HER2-negative disease only.

At the time of this analysis, 13 of 18 (72%) patients who are HIV-positive were on endocrine therapy compared with 73% (33 of 45) of patients who are HIV-negative; 1 of 45 (2%) in the HIV-negative group declined endocrine therapy and 1 of 45 (2%) was lost to follow-up. The remaining patients were deceased.

## DISCUSSION

Although there is robust literature on survival outcomes in patients who are HIV-positive and have breast cancer, this prospective cohort analysis provides novel evidence showing that patients who are HIV-positive in Botswana received less dose-intense NACT and also have lower pCR rates. These lower pCR rates would be expected to lead to reduced EFS and OS, and we report lower OS for patients who are HIV-positive compared with patients who are HIV-negative.

This prospective study has several limitations. Because of the small sample size, this study was not powered to detect significant differences in subgroup analyses; therefore, clinically meaningful differences may not be statistically significant. An important limitation is that we could not assess the relationship between RDI and pCR rates within breast cancer subtypes. Patients with hormone receptor-positive disease have lower pCR rates than other subtypes, and we could not assess the attribution of low RDI on that rate specifically. Additionally, approximately 19% (5 of 26) of patients who are HIV-positive were missing receptor status for HER2 compared with 2.4% (2 of 84) in the HIV-negative group. The differential level of missingness of HER2 data has the potential to bias our results away from the null if there were more patients with HER2 disease who did not receive targeted anti-HER2 therapy, which would have improved response. However, within patients with known HER2-positive status, pCR rates were lower for HIV-negative compared with patients living with HIV. We could not determine whether the lower pCR rates led to reduced EFS and subsequent OS. In addition, other treatment factors, including differences in adherence to adjuvant endocrine therapy and adjuvant anti-HER2 therapy, could have contributed to lower OS. However, in the multivariable analysis where hormone receptor-positive status was forced into the model, the magnitude of the association between HIV-positive and pCR was decreased suggesting that hormone receptor status may contribute partially to the lower pCR in our HIV-positive cohort. Our regression analysis within the limitations outlined suggests that RDI may be a contributor to lower pCR rates in the HIV-positive group in our cohort. Finally, among patients who are HIV-positive, CD4 and viral load data were missing for a substantial proportion of the patients. However, current Botswana treatment guidelines for HIV recommend the treat-all strategy, which aims to start anyone with a positive HIV diagnosis on treatment immediately, regardless of the CD4 count level.^20^ Subsequently, most patients who are HIV-positive have CD4 > 250 and are virally suppressed.^21^

Despite these limitations, our study has several strengths. We analyzed detailed surgical pathology data and granular chemotherapy administration data and toxicity grading which informed our analyses. Our study showed lower RDI for patients who are HIV-positive and have breast cancer, especially those on ZDV ART, which is a novel finding and an important consideration for improving outcomes in patients who are HIV-positive receiving cytotoxic chemotherapy. Although ZDV-containing therapy is no longer first-line ART in Botswana, the median duration of ART for our population was 9.8 years, suggesting that at least half of the patients started therapy before updated guidelines which recommend dolutegravir as first-line therapy. Additionally, current guidelines do recommend certain ZDV-based therapies (eg, ZDV, lamivudine, and atazanavir coformulated with ritonavir) in the list of second-line therapies to be considered in patients living with HIV who develop specific resistant mutations on first-line therapy.^22^

The OS disparity documented in our study is similar to those in large retrospective cohort analysis in the United States of patients who are HIV-positive and have breast cancer with increased all-cause mortality (HR = 4.62 [95% CI, 3.92 to 5.45]).^3^ Similar studies from SSA have shown lower OS in patients who are HIV-positive and have breast cancer compared with patients who are HIV-negative and have breast cancer.^5^ Notably, even among patients who are HIV-negative, although the 2-year OS for stage III patients was similar to rates in SSA,^23^ it was lower than 5-year OS for North American cohorts, which is 75%.^24^ The difference is likely related to lower rates of optimal therapy delivery, differences in general health care infrastructure between Botswana and North America, differences in disease biology and younger age at presentation. In addition, of note, 83% of our patient population had stage III disease, which is also higher than the proportion of patients with de novo breast cancer in a North American cohort (27% on the basis of 2012-2016 SEER data). This is likely reflective of lack of screening, breast cancer awareness, and early detection programs in Botswana, which is similar to most African countries.^25–27^

We confirmed our hypothesis that pCR rates will be lower in patients who are HIV-positive. Nietz et al showed similar findings in a South African cohort with patients who are HIV-positive less likely to achieve pCR (OR = 0.48; 95% CI, 0.27 to 0.86).^28^ A potential mechanism may be disparities in dose intensity between the two groups, which was significantly associated with pCR in the multivariable analyses. The small sample size and the differences in proportion of missing molecular status data do not allow us to conclusively account for the contributions of molecular status and RDI on pCR. Importantly, we noted that the proportion of patients receiving optimal dose intensity was low in patients who are HIV-positive and HIV-negative, which is concerning and an indicator of suboptimal therapy delivery at baseline for all patients with breast cancer regardless of HIV status. HIV-positive is further associated with even lower dose intensity of therapy represented by a larger proportion of patients receiving low dose intensity therapy and a significantly lower mean RDI. Assessment of RDI in patients with locally advanced diseased in Rwanda showed that 61.9% of those with locally advanced disease receiving NACT had RDI ≥ 0.85,^29^ compared with 44% in our cohort. These data are robust and important for assessing optimal therapy delivery and explaining disparities in outcomes; however, few countries in SSA have these data because of lack of electronic medical record systems, paper-based record keeping, and other logistical challenges.

We report a significant difference in RDI between patients who are HIV-positive and have breast cancer and patients who are HIV-negative and have breast cancer, which may contribute to lower rates of pCR and worse OS. We hypothesize that this could be partly due to toxicity in patients who are HIV-positive. In fact, although patients who are HIV-positive received significantly less dose intense therapy, toxicity rates were similar between the two groups, which suggests that patients who are HIV-positive were dose reduced in an attempt to prevent greater toxicity, although we do not have data on decision making by the providers to support that. Lower RDI may also be due to intolerance of the complete regimen among patients who are HIV-positive, evidenced by the higher but nonsignificant rate of premature treatment discontinuation in that group. Furthermore, our analysis suggests that differences in RDI may have been due to longer cycle lengths and time to treatment completion among patients who are HIV-positive compared with patients who are HIV-negative, which may also have been related to slower recovery after each cycle by patients who are HIV-positive. Furthermore, patients who are HIV-positive had more cycles with dose reductions compared with patients who are HIV-negative, which may be appropriate but also reflect a provider bias to recommend dose reduction or premature discontinuation of therapy in patients who are HIV-positive and experience toxicity compared with patients who are HIV-negative.^30^ The combined effects of higher premature discontinuation, dose reductions, and increased median duration of treatment cycle may contribute to lower RDI in patients living with HIV and in particular patients on ZDV. Since the design of the study, new National Comprehensive Cancer Network guidelines states that ZDV is contraindicated in patients who are HIV-positive initiating cytotoxic chemotherapy because of risk of causing or exacerbating myelosuppression.^31^

In conclusion, patients who are HIV-positive and have breast cancer have lower pCR rates, which may be due in part due to reduced RDI and may subsequently lead to worse OS. The frequency and magnitude of low dose intensity therapy among patients who are HIV-positive and have breast cancer highlight the challenges clinicians face in delivering optimal dose intensity treatment to this population. Although some risk factors are not modifiable, it is critical to examine implementation strategies that can specifically target modifiable risk factors associated with suboptimal therapy delivery for patients who are HIV-positive. Our next steps are to implement strategies for deimplementation of ZDV, whereby it is replaced with a safer alternative ART before initiation of cytotoxic chemotherapy. Additional considerations are the implementation of granulocyte colony-stimulating factor prophylaxis in patients living with HIV initiating AC-T or other cytotoxic chemotherapy regimens with high (≥ 20%) risk of myelosuppression. Our results highlight that these implementation strategies should be performed within a broader context of optimizing care delivery for all patients, including HIV-negative. These will include efforts to decentralize cancer care and increase breast cancer treatment access in Botswana and to improve standardization of chemotherapy administration and delivery of optimal dose intensity.

## Data Availability

Data are available upon reasonable request and will require local IRB approval or exemption in Botswana.
